# Effects of faecal microbiota transplantation on glycaemic outcomes in adults with metabolic disorders: a systematic review and meta-analysis of randomised controlled trials

**DOI:** 10.3389/fmed.2026.1983162

**Published:** 2026-10-16

**Authors:** Maryana Savytska, Viktoriya-Svitlana Halich, Liudmyla Kuzmenko, Iryna Halabitska, Iryna Kamyshna, Pavlo Petakh, Oleksandr Kamyshnyi, Nazarii Kobyliak

**Affiliations:** 1 Physiology Department, Danylo Halytsky Lviv National Medical University, Lviv, Ukraine; 2 Bogomolets National Medical University, Kyiv, Ukraine; 3 Department of Therapy and Family Medicine, I. Horbachevsky Ternopil National Medical University, Ternopil, Ukraine; 4 Department of Medical Rehabilitation, I. Horbachevsky Ternopil National Medical University, Ternopil, Ukraine; 5 Department of Biochemistry and Pharmacology, Uzhhorod National University, Uzhhorod, Ukraine; 6 Department of Microbiology, Virology, and Immunology, I. Horbachevsky Ternopil National Medical University, Ternopil, Ukraine

**Keywords:** faecal microbiota transplantation, HOMA-IR, insulin resistance, meta-analysis, metabolic syndrome, systematic review, type 2 diabetes mellitus

## Abstract

**Background:**

Faecal microbiota transplantation (FMT) may improve glucose metabolism, but findings from randomised trials and previous meta-analyses are inconsistent. We evaluated the effects of FMT on glycaemic outcomes in adults with type 2 diabetes, prediabetes, insulin resistance or related metabolic disorders.

**Methods:**

We searched PubMed and Scopus for English-language randomised controlled trials published from January 2000 to May 2026. The primary outcome was change from baseline in HOMA-IR. Secondary outcomes included HbA1c, fasting glucose and clamp-derived peripheral insulin sensitivity. Random-effects meta-analyses used Paule–Mandel estimation with Hartung–Knapp–Sidik–Jonkman confidence intervals. Sensitivity analyses evaluated comparator type, data reconstruction, the assumed baseline–follow-up correlation and individual-study influence. Risk of bias was assessed using RoB 2.

**Results:**

The searches identified 887 records, and 18 studies met the eligibility criteria. Six trials involving 120 participants included to the primary analysis. FMT did not significantly reduce HOMA-IR compared with control (MD, −1.29; 95% CI [−3.04, 0.47]; I^2^ = 72%). The sensitivity analysis combining HOMA-IR and HOMA2-IR also produced a non-significant result (MD, −1.11; 95% CI [−2.50, 0.29]). HbA1c data were sparse and were not pooled across comparator types. Clamp-derived findings suggested a possible donor-phenotype effect but remained inconclusive. The certainty of evidence was very low for HOMA-IR and HbA1c and low for peripheral insulin sensitivity.

**Conclusions:**

FMT did not demonstrate a clear benefit for glycaemic outcomes. The estimates were imprecise, heterogeneous and influenced by comparator design. Larger trials with standardised outcomes and predefined donor phenotypes are required.

## 1 Introduction

Insulin resistance, prediabetes and type 2 diabetes mellitus represent a major and growing global health burden, with approximately 589 million adults living with diabetes and 853 million projected to be affected by 2050 (1). Insulin resistance is also central to obesity, metabolic syndrome and metabolic dysfunction-associated steatotic liver disease (2, 3). Current treatments reduce cardiometabolic risk but may not fully restore insulin sensitivity, prompting investigation of additional therapeutic targets (4, 5).

Modulation of the gut microbiota is one potential approach, as microbial composition and function are linked to energy harvest, intestinal barrier integrity, immune activation, bile acid signalling and glucose metabolism (2, 6, 7). Changes in gut microbiota and microbiota-related endotoxaemia may promote low-grade inflammation and insulin resistance, while microbial metabolites, including short-chain fatty acids, secondary bile acids, branched-chain amino acid metabolites and indole derivatives, can influence pathways regulating glucose homeostasis (2, 8). However, substantial heterogeneity across populations, analytical platforms and geographical settings has limited the identification of microbial taxa consistently associated with metabolic disease (9).

Faecal microbiota transplantation (FMT) transfers a complex microbial community from a selected donor to a recipient and has been investigated both as a potential therapeutic intervention and as an experimental approach to examine the role of the gut microbiota (10). Its clinical development has occurred primarily in recurrent *Clostridioides difficile* infection, where efficacy, donor screening, preparation and safety have been addressed through systematic reviews and international guidance (11). Extension of FMT to metabolic disease remains investigational because optimal donor characteristics, administration regimens, durability of engraftment, relevant microbial targets and long-term safety have not been established (12, 13).

Systematic reviews and meta-analyses of FMT in obesity, metabolic syndrome and type 2 diabetes have reached different conclusions regarding glycaemic and cardiometabolic outcomes (14, 15). Some have reported potentially favourable changes in HOMA-IR, fasting glucose, insulin, HbA1c, body weight or lipid measures, whereas others have considered the evidence inconsistent or insufficient for clinical implementation (16, 17). Differences in eligibility criteria, comparator types, outcome definitions, follow-up periods, data transformations and statistical models may contribute to these discrepancies, despite overlap in included trials (18, 19). An umbrella review similarly found that reported FMT effects vary across diseases, outcomes and analytical approaches (17). Previous meta-analyses have combined different comparator types, although placebo, autologous FMT and active pharmacotherapy address different clinical questions (20). Glycaemic outcomes are not interchangeable: HOMA-IR and HOMA2-IR use different calculation models, whereas HbA1c, fasting glucose and clamp-derived insulin sensitivity reflect different aspects of glucose metabolism and should therefore be analysed separately (14). Treatment response may also vary with follow-up duration, administration route, treatment intensity, microbial engraftment and donor or recipient characteristics (10, 21). Most metabolic FMT trials are small, increasing statistical uncertainty (22). More conservative random-effects approaches, including Paule–Mandel estimation, HKSJ-adjusted confidence intervals and prediction intervals, may provide a more appropriate assessment of this uncertainty, particularly when effect estimates require reconstruction from incomplete summary data (23, 24).

Previous systematic reviews have therefore reported inconsistent effects of FMT on glycaemic and insulin-resistance outcomes, with important differences in population eligibility, comparator selection, outcome definitions, follow-up selection and statistical methods. The present review was designed to provide a more focused assessment of randomised evidence while accounting for these sources of heterogeneity. Because available metabolic FMT trials remain few and generally small, the objective was not to determine whether a predefined information size had been reached, but to quantify the available evidence and its statistical uncertainty using conservative random-effects methods and prespecified sensitivity analyses.

The primary objective of this systematic review and meta-analysis was to estimate the effect of allogeneic FMT on changes in HOMA-IR among adults with type 2 diabetes, prediabetes, insulin resistance or related metabolic conditions. Secondary objectives were to assess HbA1c, fasting glucose and clamp-derived peripheral insulin sensitivity, and to examine whether estimates varied according to comparator type, outcome definition, follow-up duration and analytical assumptions.

## 2 Methods

This systematic review and meta-analysis was conducted and reported in accordance with the Preferred Reporting Items for Systematic Reviews and Meta-Analyses (PRISMA 2020) statement. The study selection process is presented in the PRISMA flow diagram (Figure 1).

**Figure 1:**
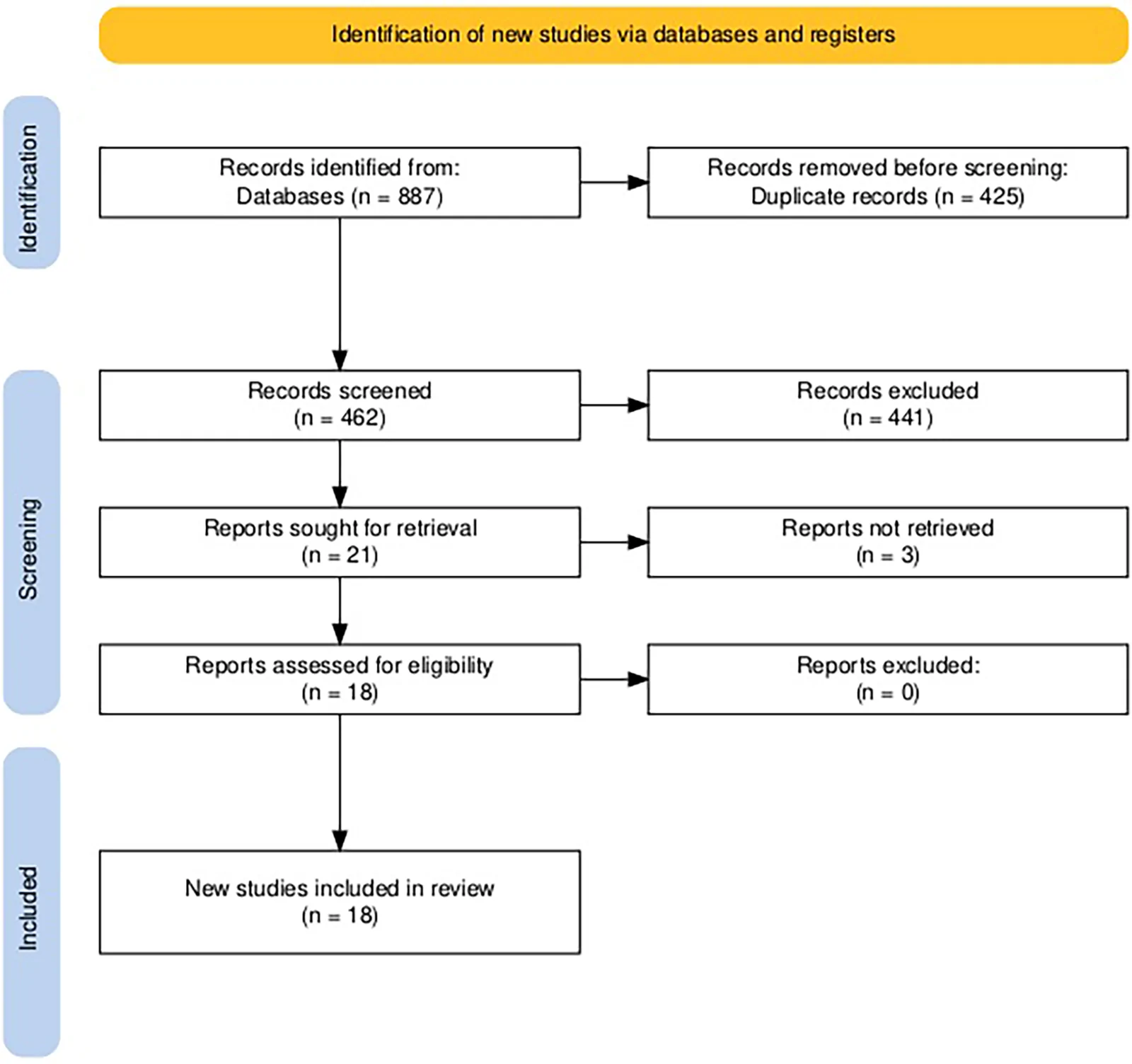
PRISMA flow diagram of study selection. Flow of records through database searching, deduplication, screening, full-text assessment and inclusion in the systematic review and meta-analysis.

### 2.1 Search strategy and study selection

We searched PubMed and Scopus for studies published from January 2000 to May 2026. The search used MeSH and free-text terms in PubMed and corresponding title, abstract and keyword terms in Scopus to identify randomised controlled trials of faecal microbiota transplantation in type 2 diabetes, prediabetes, insulin resistance and related metabolic disorders. The search was restricted to English-language publications. We also screened the reference lists of eligible studies and relevant systematic reviews to identify additional reports.

The complete electronic search strategies, including database-specific controlled vocabulary, free-text terms, Boolean operators, field restrictions and search dates, are provided in Supplementary Table S1. The strategy was developed around three main concepts: faecal microbiota transplantation, metabolic disorders or insulin resistance, and randomised controlled trials. Search terms were adapted to the indexing structure of each database.

PubMed and Scopus were selected as the primary biomedical and multidisciplinary bibliographic databases because together they provide broad coverage of biomedical, clinical and interdisciplinary literature relevant to FMT and metabolic disease. To reduce the possibility of missing eligible studies, reference lists of all included studies and relevant systematic reviews were additionally screened.

All retrieved records were imported into a single EndNote X9 library. Duplicates were identified using DOI and PMID where available. Records without these identifiers were compared using normalised titles after conversion to lowercase and removal of punctuation and excess spacing. Potential duplicates were manually verified by comparing the title, first author, publication year and journal. Two reviewers independently screened titles and abstracts and subsequently assessed the full texts of potentially eligible reports. Disagreements were resolved by consensus, with adjudication by a third reviewer when required. Reasons for full-text exclusion were recorded, and the study-selection process was summarised in a PRISMA 2020 flow diagram.

### 2.2 Protocol and reporting framework

The protocol for this systematic review and meta-analysis was not prospectively registered in PROSPERO or another systematic review registry. The review was conducted according to a prespecified methodological framework developed before data extraction and statistical analysis. The absence of prospective registration is acknowledged as a limitation.

### 2.3 Eligibility criteria

Studies were eligible if they met the following criteria: (1) study design: randomised controlled trials; (2) participants: adults aged ≥18 years with type 2 diabetes, prediabetes, insulin resistance or a metabolic condition associated with impaired glucose metabolism, including type 2 diabetes mellitus, prediabetes, impaired glucose tolerance, metabolic syndrome, obesity or metabolic dysfunction-associated steatotic liver disease, provided that an eligible glycaemic or insulin-resistance outcome was assessed; (3) intervention: allogeneic FMT administered by any route; (4) comparator: placebo or sham procedure, autologous FMT, no-FMT control or active pharmacotherapy; and (5) outcomes: HOMA-IR, HOMA2-IR, HbA1c, fasting glucose or peripheral insulin sensitivity measured as glucose disposal rate (Rd) during a hyperinsulinaemic–euglycaemic clamp. Non-randomised studies, observational studies, animal experiments, reviews and studies without an eligible comparator were excluded.

### 2.4 Data extraction

Two reviewers independently extracted data from each included study using a standardised workbook. Disagreements were resolved through discussion and, when necessary, consultation with a third reviewer. The extracted data included study design, participant characteristics, sample size, metabolic condition, intervention and comparator regimens, FMT administration route, donor phenotype, follow-up duration and reported glycaemic outcomes. For each outcome, we recorded baseline and follow-up values, change scores, measures of dispersion, adjusted between-group estimates and confidence intervals.

When multiple eligible estimates were reported, we selected the prespecified primary time point and preferentially used adjusted between-group estimates. Studies lacking sufficient data to calculate an effect estimate and its standard error were included in the qualitative synthesis but excluded from the quantitative meta-analysis.

### 2.5 Outcome measures

The primary outcome was the change from baseline in HOMA-IR. Secondary outcomes included changes in HbA1c, fasting blood glucose and clamp-derived glucose disposal rate (Rd). HOMA-IR and HOMA2-IR were analysed separately because they are calculated using different models. HOMA2-IR data were excluded from the primary analysis and included only in a sensitivity analysis. HbA1c results were reported separately according to comparator type and were not pooled across sham and active-drug controls. Fasting-glucose outcomes were summarised narratively when quantitative pooling was not appropriate. Rd was analysed separately as a direct measure of peripheral insulin sensitivity.

When multiple follow-up assessments were available, data were extracted from the time point defined as primary in the original trial. If the original trial did not specify a primary time point, the longest post-intervention assessment with sufficient data for the prespecified outcome was selected. This approach was chosen to capture the most sustained available effect of FMT rather than preferentially selecting an early response, given that microbiome engraftment and downstream metabolic effects may evolve over time. Shorter-term assessments were retained for descriptive and sensitivity analyses when relevant.

### 2.6 Risk-of-bias assessment

Two reviewers independently assessed the risk of bias in each included randomised trial using the Cochrane Risk of Bias tool, version 2 (RoB 2) (25). The assessment focused on the effect of assignment to the intervention and was performed for the outcome and follow-up time point included in the synthesis.

Five domains were evaluated: the randomisation process, deviations from intended interventions, missing outcome data, outcome measurement and selection of the reported result. Each domain was rated as “low risk,” “some concerns” or “high risk,” and the overall judgement was determined according to the RoB 2 algorithm. Disagreements were resolved through discussion or consultation with a third reviewer. Risk-of-bias judgements were summarised in a traffic-light plot, with supporting rationales provided in the Supplementary Materials (26).

### 2.7 Data preparation and management of complex trial designs

Continuous outcomes were expressed as between-group mean differences in change from baseline in accordance with the Cochrane Handbook (27). When change-score standard deviations were not reported, they were estimated from baseline and follow-up standard deviations using a correlation coefficient of 0.50. Correlation coefficients of 0.25 and 0.75 were examined in sensitivity analyses.

Means and standard deviations were estimated from medians, interquartile ranges or ranges using the methods of Wan et al. and Luo et al. (28, 29). Studies requiring these conversions were identified as reconstructed and evaluated in separate sensitivity analyses. When a mean difference and 95% confidence interval were reported without a standard error, the standard error was derived from the confidence-interval width using standard inverse-variance methods. Data were digitised from published figures only when numerical results were otherwise unavailable. The source figure, extracted values and underlying assumptions were documented in the supplementary workbook, and digitised estimates were treated as approximate.

For multi-arm trials, eligible intervention groups were combined and shared control groups were divided to avoid double-counting. In factorial trials, groups differing only in the co-intervention were combined to estimate the overall effect of FMT. FMT alone and FMT combined with active pharmacotherapy were analysed as separate comparisons.

### 2.8 Statistical analysis

All analyses were performed in R using inverse-variance random-effects models. The primary meta-analysis was restricted to conventional HOMA-IR outcomes. Between-study variance was estimated using the Paule–Mandel method, and 95% confidence intervals were calculated using the Hartung–Knapp–Sidik–Jonkman adjustment (24, 30, 31). DerSimonian–Laird models with Wald confidence intervals were performed as a sensitivity analysis for comparison with previous meta-analyses.

Statistical heterogeneity was assessed using Cochran's Q and quantified using *τ*^2^ and I^2^. Ninety-five per cent prediction intervals were calculated for analyses including at least three studies. Single-study effects were presented with Wald confidence intervals, and analyses containing two studies were considered exploratory. HbA1c results were reported separately by comparator type and were not pooled. The two lean-donor Rd studies were summarised using a descriptive DerSimonian–Laird random-effects model with a Wald confidence interval. The digitised Rd comparison involving metabolically impaired donors was presented separately.

Sensitivity analyses examined the effects of combining HOMA-IR with HOMA2-IR, excluding reconstructed estimates, varying the baseline–follow-up correlation and excluding the most influential study. The primary analysis was repeated using correlation coefficients of 0.25, 0.50 and 0.75. Comparator-specific analyses were conducted separately for placebo or sham, autologous FMT and active pharmacotherapy.

Study influence was assessed using leave-one-out analysis, standardised residuals, study-specific contributions to Cochran's Q, changes in the pooled estimate after study omission and a Baujat plot. Peripheral insulin-sensitivity findings were also examined according to donor metabolic phenotype.

Funnel plots and statistical tests for small-study effects were not performed because fewer than ten studies were available for each outcome. The certainty of evidence was assessed using GRADE across the domains of risk of bias, inconsistency, indirectness, imprecision and publication bias (32). Certainty was rated as high, moderate, low or very low, with reasons for downgrading documented for each outcome.

## 3 Results

### 3.1 Study selection

The database searches identified 887 records, including 210 from PubMed and 677 from Scopus. After removal of 425 duplicates, 462 records underwent title and abstract screening, of which 441 were excluded. Full texts were sought for 21 reports, but three could not be retrieved. All 18 retrieved reports met the eligibility criteria and were included in the systematic review. No reports were excluded following full-text assessment. Six trials involving 120 participants contributed to the primary HOMA-IR meta-analysis. The study-selection process is presented in Figure 1.

These broader metabolic conditions were included because FMT has been investigated as a microbiome-directed intervention in populations with insulin resistance or impaired glucose metabolism even when metabolic disorders was not the formal diagnostic inclusion criterion. Eligibility therefore depended on the metabolic phenotype evaluated in the trial and the availability of prespecified glycaemic or insulin-resistance outcomes rather than on the diagnostic label alone.

### 3.2 Study characteristics

Eighteen reports of randomised controlled trials published between 2012 and 2025 were included (33–50). The studies enrolled adults with obesity, metabolic syndrome, insulin resistance, type 2 diabetes mellitus, or NAFLD/MASLD. Most trials had small sample sizes, and follow-up duration varied across studies.

FMT was administered by upper gastrointestinal infusion, colonoscopy, rectal enema, or oral capsules. Comparators included placebo or sham, autologous FMT, and active treatment with metformin. Most studies used stool from lean or metabolically healthy donors. De Groot et al. compared FMT from donors following Roux-en-Y gastric bypass with FMT from donors with metabolic syndrome (36). Several trials evaluated FMT together with dietary intervention or fibre supplementation (35, 41, 42, 44, 46).

HOMA-IR was the most frequently reported glycaemic outcome. Other outcomes included HOMA2-IR, HbA1c, fasting glucose, and clamp-derived glucose disposal rate. Six trials comprising 120 participants contributed to the primary HOMA-IR meta-analysis (35, 37–39, 47, 50). Craven et al. and Ghorbani et al. were included in the qualitative synthesis but excluded from meta-analysis because the reported data were insufficient to derive a between-group effect estimate and its standard error (48, 49). Detailed study characteristics are presented in Table 1 and Supplementary Table S2.

**Table 1:** Characteristics of the included studies.

Study	Population	Randomised, n^a^	Intervention and comparator	Administration and follow-up	Glycaemic outcomes
Vrieze et al., 2012 (33)	Men with obesity and metabolic syndrome	18	Allogeneic FMT from lean donors vs. autologous FMT	Single nasoduodenal infusion; 6 weeks	Clamp-derived peripheral insulin sensitivity (Rd)
Kootte et al., 2017 (34)	Men with metabolic syndrome	38	Allogeneic FMT from lean donors vs. autologous FMT	Single nasoduodenal infusion; 6 and 18 weeks	Clamp-derived peripheral insulin sensitivity (Rd)
Koopen et al., 2021 (35)	Men with metabolic syndrome following a Mediterranean-diet run-in	24	Allogeneic FMT from lean donors vs. autologous FMT; Mediterranean diet in both groups	Single nasoduodenal infusion; 6 weeks	HOMA-IR, fasting glucose and clamp-derived Rd
de Groot et al., 2020 (36)	Men with obesity, metabolic syndrome and insulin resistance	22	FMT from post-Roux-en-Y gastric bypass donors vs. donors with metabolic syndrome	Single upper-gastrointestinal infusion; 2 weeks, with additional assessments at 8 weeks	Clamp-derived peripheral and hepatic insulin sensitivity
Yu et al. (FMT-TRIM), 2020 (37)	Adults with obesity and mild-to-moderate insulin resistance	24	Allogeneic FMT from healthy lean donors vs. placebo	Weekly oral capsules for 6 weeks; 12-week follow-up	HOMA-IR, HbA1c and clamp-derived insulin sensitivity
Gómez-Pérez et al., 2024 (38)	Adults with type 2 diabetes and marked insulin resistance	21^b^	Pooled lean-donor FMT vs. placebo or probiotic	Oral lyophilised capsules; 12 weeks	HOMA-IR, fasting glucose and HbA1c
Wu et al., 2023 (39)	Adults with newly diagnosed type 2 diabetes	31^c^	FMT alone, FMT plus metformin, or metformin alone	Nasojejunal infusion; 4 weeks	HOMA-IR, fasting glucose and HbA1c
da Ponte Neto et al., 2023 (40)	Adults with obesity and metabolic syndrome	32	Allogeneic FMT vs. saline sham	Single upper-gastrointestinal endoscopic infusion; assessments up to 1 year	HbA1c and fasting glucose
Mocanu et al., 2021 (41)	Adults with severe obesity and metabolic syndrome	70	Factorial comparison of pooled-donor FMT or placebo, with or without fibre supplementation	Single-dose oral capsules; 12 weeks	HOMA2-IR, fasting glucose, insulin and HbA1c
Ng et al., 2022 (42)	Adults with obesity and type 2 diabetes	61	FMT plus lifestyle intervention, FMT alone, or sham plus lifestyle intervention	Repeated duodenal administration every 4 weeks through week 12; 24-week follow-up	Fasting glucose, insulin and HbA1c
Allegretti et al., 2020 (43)	Adults with obesity without diabetes or metabolic syndrome	22	Oral FMT from a lean donor vs. placebo capsules	Induction and maintenance capsule doses; 26-week follow-up	Fasting glucose, insulin and HbA1c as secondary outcomes
Rinott et al., 2021 (44)	Adults with abdominal obesity or dyslipidaemia following diet-induced weight loss	90	Diet-modulated autologous FMT vs. placebo	Oral frozen capsules during the weight-regain phase; 14 months	HOMA-IR and other glycaemic measures as secondary outcomes
Smits et al., 2018 (45)	Men with metabolic syndrome	20	Allogeneic FMT from lean vegan donors vs. autologous FMT	Single nasoduodenal infusion; 2 weeks	Metabolic and glycaemic measures as secondary outcomes
Su et al., 2022 (46)	Adults with type 2 diabetes	16^d^	Healthy-donor FMT plus structured diet vs. structured diet alone	Oral capsules on days 1, 8 and 15; 90 days	Fasting glucose and HbA1c
Groenewegen et al., 2025 (47)	Adults with metabolic dysfunction-associated steatotic liver disease	20	Allogeneic FMT vs. autologous FMT	Repeated duodenal administration; 12 weeks	HOMA-IR and oral glucose-tolerance outcomes
Craven et al., 2020 (48)	Adults with non-alcoholic fatty liver disease	21	Allogeneic FMT vs. autologous FMT	Single duodenal administration; 6-month follow-up	HOMA-IR
Ghorbani et al., 2023 (49)	Adults with severe obesity and insulin resistance	28	Allogeneic FMT from healthy lean donors vs. autologous FMT	Single colonoscopic administration into the caecum; 3 months	HOMA-IR and HbA1c
Piwchan et al., 2025	Adults with metabolic syndrome and moderate-to-severe insulin resistance	18	Allogeneic FMT from healthy donors vs. sham	Rectal enema; 6- and 12-week assessments	HOMA-IR, fasting glucose and HbA1c

a Sample sizes refer to the total randomised population unless otherwise specified.

b The FMT–placebo contrast included 14 participants.

c The primary FMT–metformin contrast included 21 participants.

d Sixteen participants were randomised and 13 completed follow-up.

### 3.3 Study characteristics

Risk-of-bias assessments are presented in Figure 2. Four studies were judged to be at low overall risk of bias (35, 37, 40, 47). Thirteen studies raised some concerns (33, 34, 36, 38, 39, 41–45, 48–50), and one study was judged to be at high risk of bias (46).

**Figure 2:**
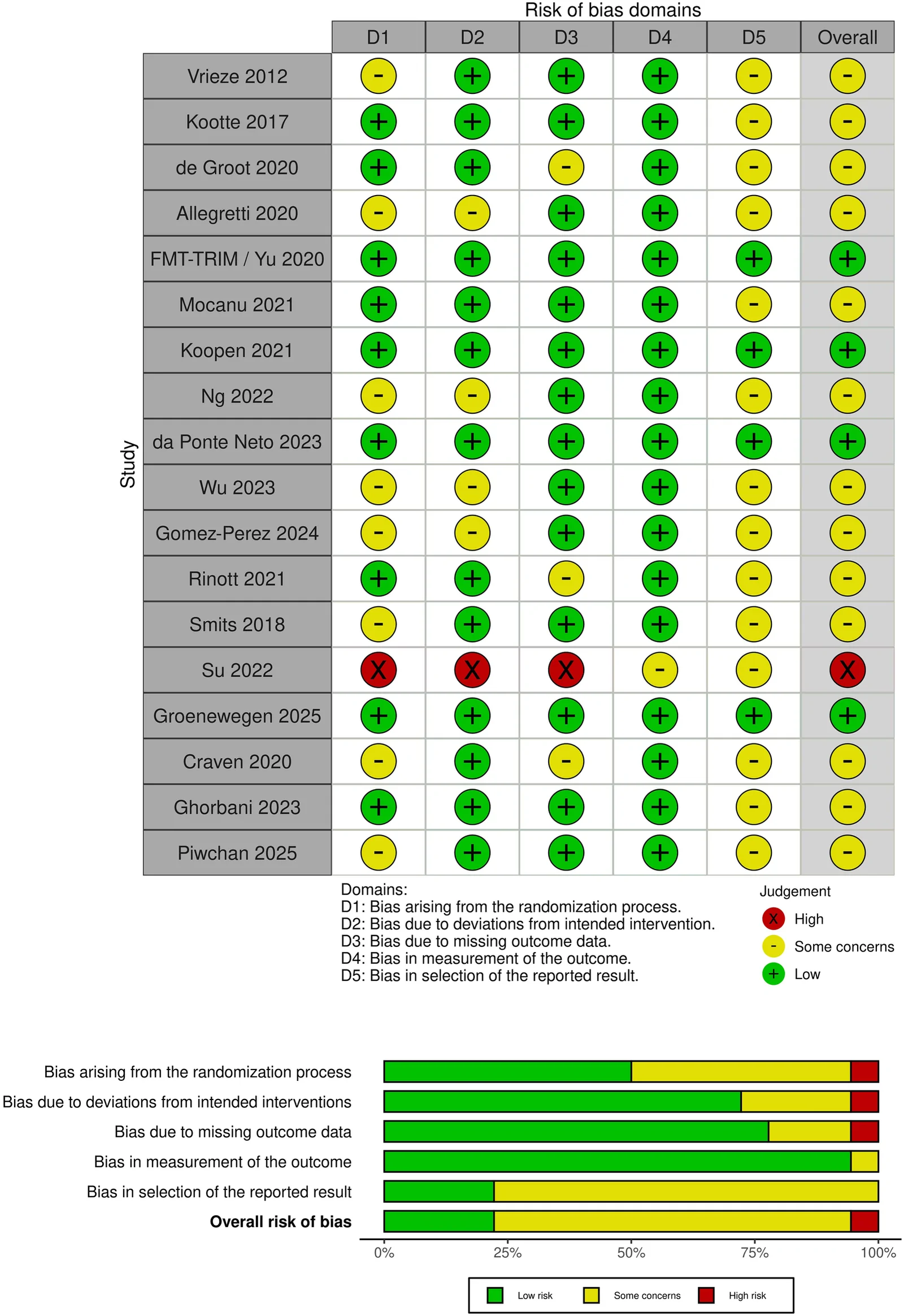
Risk of bias in included trials. Domain-level and overall RoB 2 judgements for each included randomised trial.

Concerns most frequently related to selection of the reported result, affecting 14 of the 18 studies. Concerns regarding the randomisation process, deviations from intended interventions and missing outcome data were identified in nine, five and four studies, respectively. Outcome measurement was judged to be at low risk of bias in 17 studies. Su et al. was judged to be at high risk because of limitations in randomisation, the open-label design, attrition and incomplete outcome reporting (46).

### 3.4 HOMA-IR

The primary analysis included six trials reporting conventional HOMA-IR outcomes (*N* = 120) (35, 37–39, 47, 50). FMT was not associated with a significant reduction in HOMA-IR compared with control (MD −1.29, 95% CI −3.04 to 0.47; 95% PI −5.42 to 2.85; I^2^ = 72%; *τ*^2^ = 1.75; Figure 3, Table 2).

**Figure 3:**
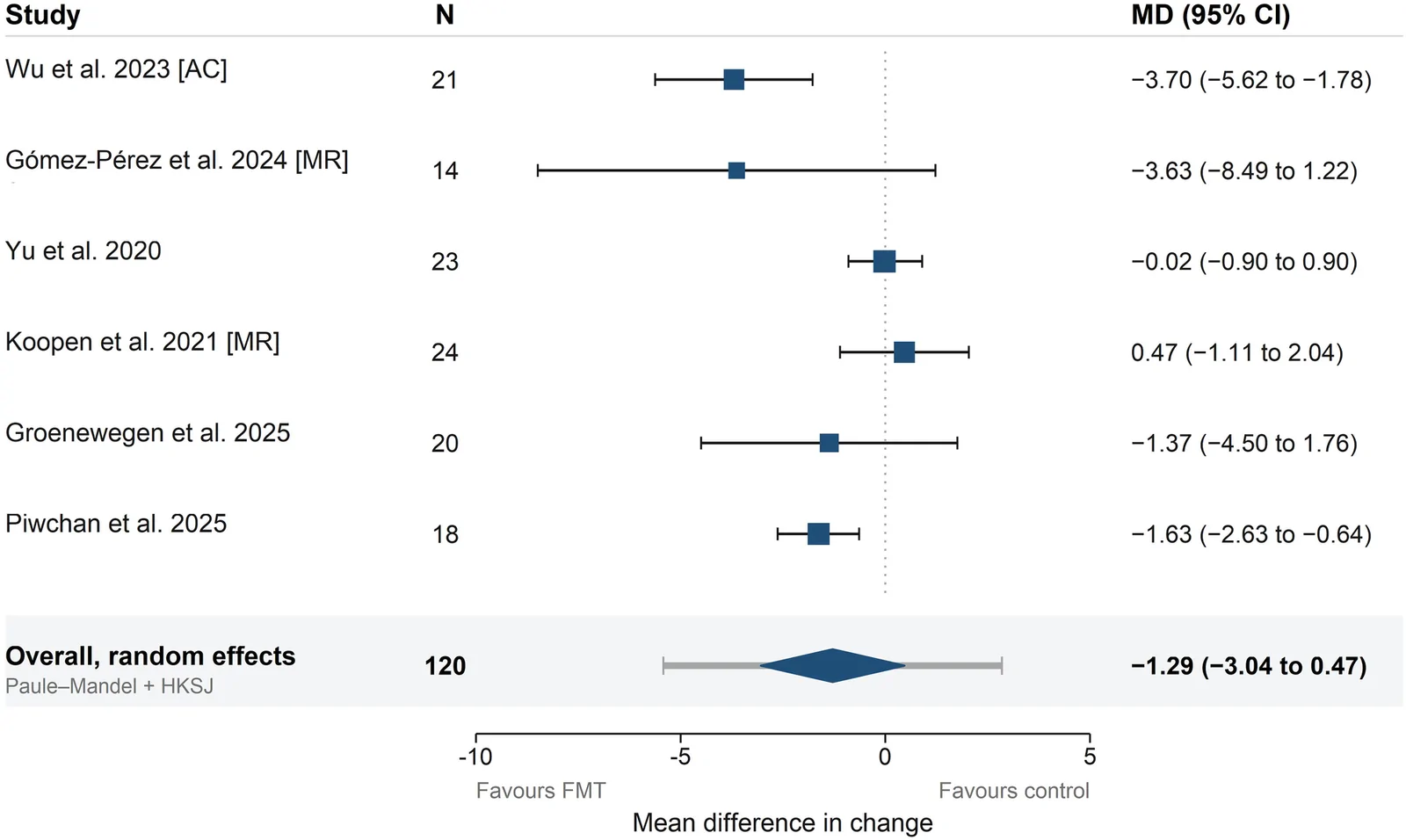
Effect of FMT on HOMA-IR. Study-specific effects and pooled mean differences in HOMA-IR change; negative values favour FMT.

**Table 2:** Primary and secondary analyses of HOMA-IR.

Analysis	k	N	MD (95% CI)	I^2^
Primary HOMA-IR analysis	6	120	−1.29 (−3.04 to 0.47)	72%
Scale-mixed HOMA-IR and HOMA2-IR	7	190	−1.11 (−2.50 to 0.29)	68%
Placebo or sham comparator	3	55	−1.05 (−4.38 to 2.29)	71%
Autologous FMT comparator	2	44	0.07 (−9.56 to 9.69)	5%
Active-drug comparator	1	21	−3.70 (−5.62 to −1.78)	—
Primary analysis excluding Wu et al.	5	99	−0.69 (−2.16 to 0.78)	57%

The placebo- or sham-controlled analysis included three trials (*N* = 55) and showed an MD of −1.05 (95% CI −4.38 to 2.29; *I*^2^ = 71%) (37, 38, 50). The pooled estimate for the two autologous-FMT-controlled trials was 0.07 (95% CI −9.56 to 9.69; *I*^2^ = 5%) (35, 47). The single metformin-controlled trial favoured FMT (MD −3.70, 95% CI −5.62 to −1.78), but the comparison was affected by a marked baseline imbalance in HOMA-IR (39). Adding the HOMA2-IR trial produced a scale-mixed analysis of seven trials (*N* = 190) (41). The pooled estimate remained non-significant (MD −1.11, 95% CI −2.50 to 0.29; *I*^2^ = 68%).

### 3.5 Peripheral insulin sensitivity and HbA1c

Two trials assessed clamp-derived peripheral insulin sensitivity after FMT from lean donors (*N* = 56) (33, 34). The descriptive pooled estimate favoured FMT but was imprecise (MD 5.42 μmol/kg/min, 95% CI −1.43 to 12.28; *I*^2^ = 11%; Figure 4).

**Figure 4:**
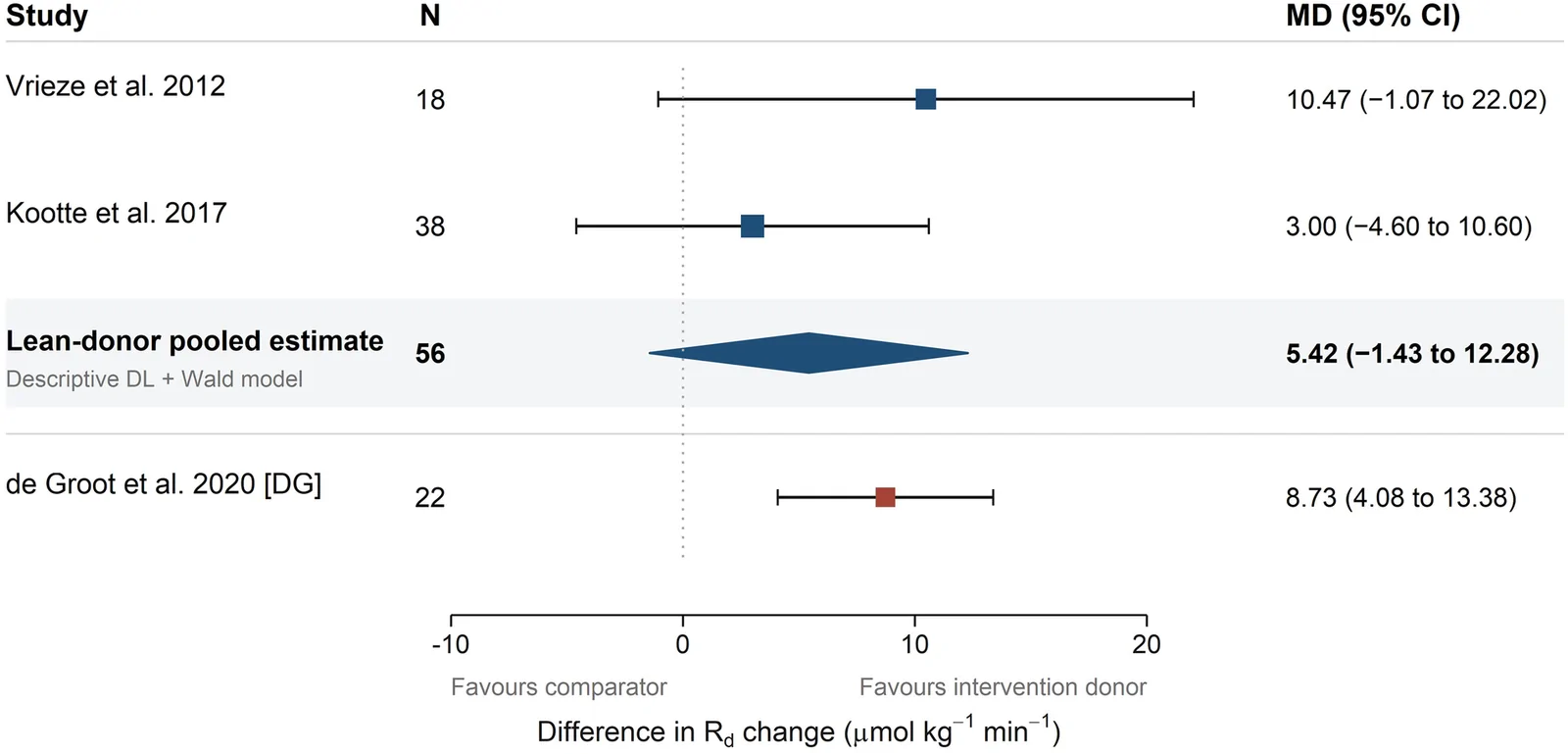
Peripheral insulin sensitivity by donor phenotype. Between-group differences in clamp-derived glucose disposal rate, presented separately by donor metabolic phenotype.

HbA1c findings were reported separately by comparator type. Da Ponte Neto et al. found no difference between FMT and sham (MD 0.14%, 95% CI −1.09 to 1.37) (40). Wu et al. reported a reduction relative to metformin (MD −1.91%, 95% CI −3.31 to −0.51), although this estimate was affected by the baseline imbalance between groups (39). Gómez-Pérez et al. reported a small within-group increase in HbA1c after FMT (+0.25%; *P* = 0.041) (38). These results were not pooled because the comparator designs and reported effect measures differed (Figure 5).

**Figure 5:**
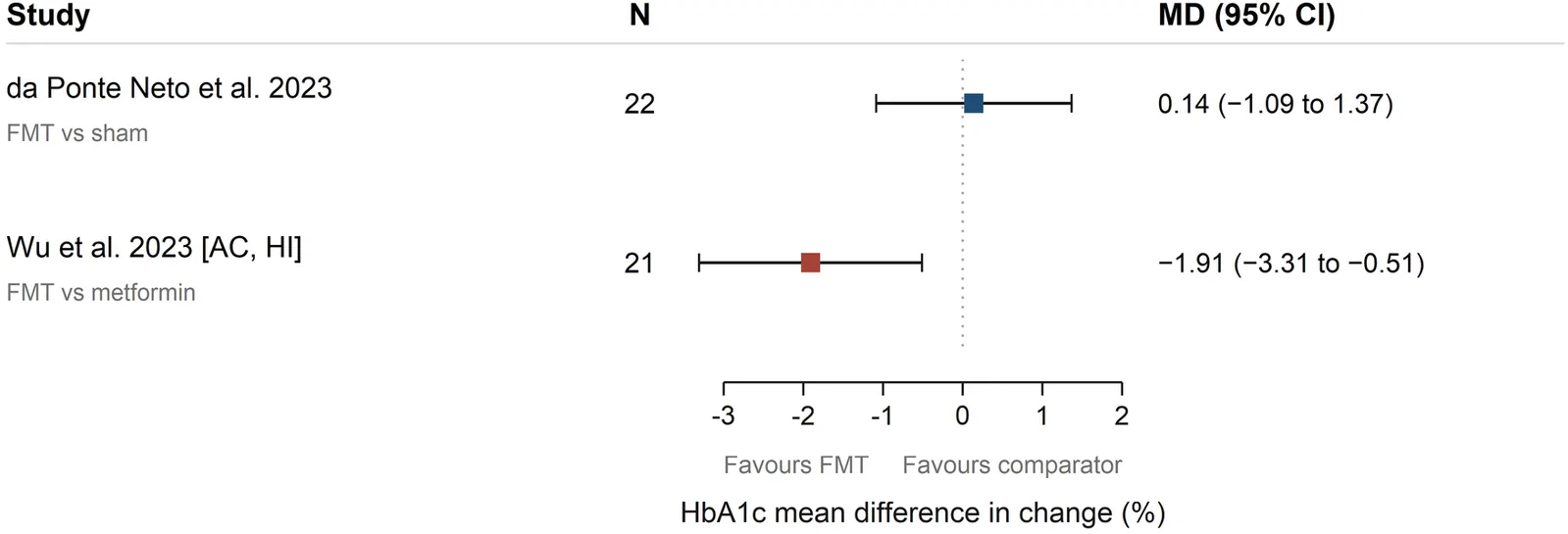
Effect of FMT on HbA1c. Comparator-specific differences in HbA1c change; estimates were presented separately and not pooled.

### 3.6 Sensitivity and influence analyses

The HOMA-IR result was consistent across baseline–follow-up correlations of 0.25–0.75. Pooled MDs ranged from −1.36 to −1.22. In the scale-mixed analysis, the DerSimonian–Laird model yielded an MD of −1.07 (95% CI −2.07 to −0.06), whereas the Paule–Mandel–HKSJ model yielded an MD of −1.11 with a wider confidence interval (95% CI −2.50 to 0.29).

Wu et al. had the greatest influence on the primary pooled estimate (39). Exclusion of this study reduced the pooled effect from −1.29 to −0.69 and reduced heterogeneity from 72% to 57% (MD −0.69, 95% CI −2.16 to 0.78) (Figure 6).

**Figure 6:**
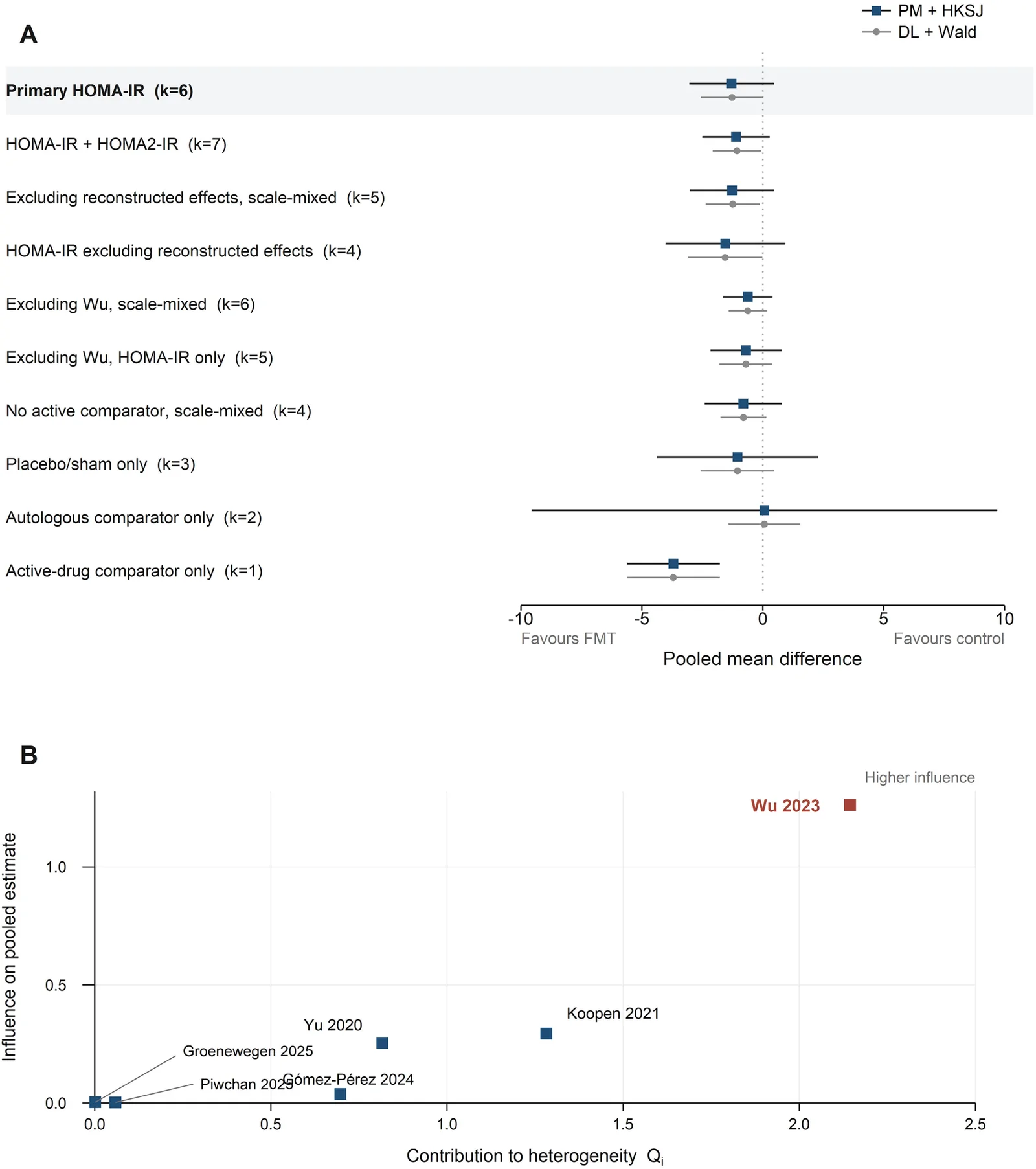
Robustness and influence analyses. **(A)** Pooled estimates across alternative analytic specifications. **(B)** Baujat plot showing each study's contribution to heterogeneity and influence on the pooled effect.

### 3.7 Certainty of evidence

The certainty of evidence was rated very low for HOMA-IR because of risk of bias, substantial heterogeneity and imprecision. Certainty was also very low for HbA1c because each comparator-specific estimate was based on a single small trial. Evidence for the lean-donor Rd analysis was rated low because only two trials were available and the pooled estimate was imprecise. The comparison according to donor metabolic phenotype was considered hypothesis-generating and was not formally graded.

## 4 Discussion

This systematic review did not demonstrate a significant improvement in HOMA-IR following FMT. The primary analysis included six trials and 120 participants and yielded a mean difference of −1.29 (95% CI −3.04 to 0.47). The scale-mixed analysis also showed method-dependent results. The conventional DerSimonian–Laird model produced a statistically significant estimate, whereas the Paule–Mandel model with HKSJ inference did not. This difference indicates that the apparent benefit was sensitive to the analytical method and the inclusion of HOMA2-IR.

The present findings are more conservative than several previous meta-analyses. Early reviews reported short-term improvements in HbA1c, fasting glucose, insulin resistance or selected cardiometabolic outcomes after FMT (14, 15). Subsequent meta-analyses also reported favourable pooled effects on fasting glucose or HOMA-IR (18, 19, 51). However, these estimates were based on small and overlapping trial sets.

Differences in eligibility criteria, comparator classification, follow-up selection and outcome handling may explain the discordant findings. Some reviews combined sham, autologous FMT and active-treatment comparators. Others combined HOMA-IR and HOMA2-IR or included pre–post changes without an independent control contrast.

A cardiometabolic meta-analysis found limited short-term changes and no consistent clinically important improvement in glucose regulation or insulin resistance (52). An umbrella review found no convincing benefit for metabolic syndrome and rated most associations as low- or very-low-certainty evidence (17). A broader umbrella review found that reliable evidence for FMT remained concentrated in recurrent *Clostridioides difficile* infection and selected gastrointestinal disorders (53).

Meta-analyses of microbiome-directed treatments have reported small improvements in fasting glucose and other metabolic markers (16, 54, 55). These reviews combined FMT with probiotics, prebiotics, synbiotics, dietary interventions or microbial metabolites. Findings from trials outside the primary adult metabolic disorders pool is also consistent with uncertainty. The Gut Bugs trial found no improvement in BMI or insulin sensitivity after encapsulated multi-donor FMT in adolescents with obesity, despite measurable changes in microbiome composition (56). A secondary analysis confirmed substantial donor-strain engraftment but also demonstrated marked variation between donors and recipients (57). Similarly, lean-donor FMT administered before bariatric surgery did not improve weight loss compared with autologous FMT (58).

Sterile faecal filtrate transplantation altered bacteriophage and microbial profiles without significantly improving glucose metabolism in adults with metabolic syndrome (59). At four-year follow-up, participants in the Gut Bugs trial had favourable differences in waist circumference, body fat and metabolic syndrome severity, but no significant improvement in glucose markers (60).

Microbial engraftment does not necessarily indicate clinical benefit. Donor-derived bacteria and bacteriophages may persist after FMT without improving glycaemic outcomes (57, 60–62). Engraftment is also influenced by donor composition, recipient microbiota, diet, inflammation and bile-acid profiles (63–67). Its clinical relevance may vary across donors, recipients and disease settings.

The metabolic effects of FMT remain biologically plausible. Short-chain fatty acids, bile acids and specific bacterial taxa may influence intestinal barrier function, incretin secretion and insulin sensitivity (68–72). However, findings from microbiome-directed interventions cannot confirm the clinical efficacy of FMT.

However, the microbiome assessments across trials were heterogeneous, and most studies were not designed to link specific microbial taxa or functional pathways to changes in glycaemic outcomes. Therefore, microbiome findings should be considered mechanistic or exploratory rather than evidence that specific microbial changes mediate the metabolic effects of FMT.

Comparator design may also explain heterogeneity. Metformin alters the gut microbiome and may partly exert its metabolic effects through microbial pathways (73). Trials comparing FMT plus metformin with metformin alone therefore estimate an adjunctive effect and should be analysed separately from sham- or autologous-FMT-controlled trials.

## 5 Clinical implications

Current evidence does not support the routine use of FMT for improving glycaemic control. The pooled HOMA-IR estimate was imprecise, heterogeneous and sensitive to one active-comparator study. Evidence for HbA1c and clamp-derived insulin sensitivity was insufficient for a clinical recommendation.

FMT should currently be used for metabolic disorders only in clinical trials. Current consensus documents recommend research-based use for indications outside C. difficile infection because efficacy and long-term safety stay uncertain (11, 74). This position is aligns with recent expert assessment of microbiome-based interventions in diabetes (75).

Future trials should use a single predefined comparator. Sham-controlled trials should assess biological efficacy. Autologous-FMT trials should assess efficacy against a procedural and biological control. Active-drug trials should be reported as comparative or adjunctive treatment studies.

HOMA-IR and HOMA2-IR should be analysed separately. Trials should report adjusted between-group effects, complete dispersion measures and a predefined primary time point. Clamp-derived insulin sensitivity should be included in adequately powered mechanistic subsets.

Donor metabolic phenotype should be prospectively defined. Recipient characteristics, diet, medication and baseline microbiome composition should also be recorded. Microbial engraftment should be accompanied by functional measurements such as metabolomics, short-chain fatty acids and bile-acid profiles (53, 64–66).

Safety monitoring remains essential. Transmission of drug-resistant organisms has been documented after FMT (76). Future metabolic trials should use validated stool-bank procedures, donor quarantine, repeat pathogen screening and long-term adverse-event surveillance (12). Antimicrobial-resistance genes and horizontal gene transfer should also be monitored when metagenomic data are available (75).

## 6 Limitations

This review has several limitations. The primary meta-analysis included only six trials and 120 participants. Comparator-specific analyses contained one to three trials and were underpowered.

An additional limitation is that the review protocol was not prospectively registered in PROSPERO or another systematic review registry. Although the methodological framework was prespecified before data extraction and statistical analysis, the absence of prospective registration may reduce transparency regarding the timing of protocol decisions and increases the potential for selective outcome or analytical choices.

The literature search was conducted in PubMed and Scopus and was supplemented by reference-list screening. Additional databases, including regional bibliographic sources and Google Scholar, were not systematically searched. Therefore, although the combination of database searching and reference-list screening was designed to reduce the risk of missed studies, some eligible reports may not have been identified. This possibility should be considered when interpreting the completeness of the evidence base.

The included populations differed in metabolic condition, baseline insulin resistance, co-interventions, FMT route, dose, donor phenotype and follow-up duration. These differences limited the interpretation of the pooled average effect. They also prevented reliable meta-regression or formal assessment of treatment-effect modifiers.

Some effect estimates required conversion from medians or reconstruction of change-score standard deviations. The assumed baseline–follow-up correlation had little effect on the direction of the primary estimate, but residual uncertainty remained. The donor-phenotype comparison was digitised from a published figure and should be considered approximate.

Several eligible trials did not provide sufficient data for quantitative synthesis. HbA1c and clamp-derived outcomes were reported by few studies. The available evidence was therefore insufficient to assess the durability of any metabolic effect. Publication bias cannot be excluded. Restriction to English-language reports may also have language bias.

Follow-up duration varied substantially across trials, and the available assessment times were not harmonised across studies. Although the prespecified extraction rule prioritised the primary trial endpoint and, when this was not defined, the longest available post-intervention assessment, differences in treatment duration and follow-up may still contribute to clinical heterogeneity and limit conclusions regarding the durability of FMT effects.

## 7 Conclusions

FMT did not produce a statistically robust improvement in HOMA-IR. The estimate was heterogeneous, imprecise and sensitive to comparator design and statistical method. Evidence for HbA1c and peripheral insulin sensitivity remained insufficient. Importantly, the available microbiome analyses were heterogeneous and were not sufficiently standardised or consistently linked to specific microbial taxa, functional pathways or metabolic outcomes to establish a microbiome-mediated mechanism of glycaemic effects. Donor phenotype and recipient microbiome characteristics remain plausible sources of treatment heterogeneity, but their clinical relevance has not been established. Adequately powered, prospectively registered trials integrating clinical outcomes with standardised microbiome, metagenomic and functional analyses are required before FMT can be considered for metabolic disorders.

## Data Availability

The original contributions presented in the study are included in the article/Supplementary Material, further inquiries can be directed to the corresponding author.
