# Supplementary material for: Effects of faecal microbiota transplantation on glycaemic outcomes in adults with metabolic disorders: a systematic review and meta-analysis of randomised controlled trials
Source: Front Med (Lausanne). 2026 Oct 16;13:1983162. doi: 10.3389/fmed.2026.1983162 (PMC13719402; doi:10.3389/fmed.2026.1983162)
Supplement: Supplementary file 1 [file Table1.docx]

**Supplementary Table S1.** Electronic search strategies

| **Database** | **Search details** |
| --- | --- |
| PubMed | Search date: 31 May 2026; coverage: 1 January 2000–31 May 2026; language: English |
| Scopus | Search date: 31 May 2026; coverage: 1 January 2000–31 May 2026; language: English |

**PubMed**

**(**

**"Fecal Microbiota Transplantation"[Mesh]**

**OR "fecal microbiota transplant*"[tiab]**

**OR "faecal microbiota transplant*"[tiab]**

**OR "fecal microbiome transplant*"[tiab]**

**OR "faecal microbiome transplant*"[tiab]**

**OR "fecal microbial transplant*"[tiab]**

**OR "faecal microbial transplant*"[tiab]**

**OR "stool transplant*"[tiab]**

**OR "intestinal microbiota transfer*"[tiab]**

**OR (transfer*[tiab] AND intestinal[tiab] AND microbiota[tiab])**

**OR "microbiota transfer therap*"[tiab]**

**)**

**AND**

**(**

**"Diabetes Mellitus, Type 2"[Mesh]**

**OR "Prediabetic State"[Mesh]**

**OR "Insulin Resistance"[Mesh]**

**OR "Metabolic Syndrome"[Mesh]**

**OR "Obesity"[Mesh]**

**OR "Glucose Intolerance"[Mesh]**

**OR "Hyperglycemia"[Mesh]**

**OR "Non-alcoholic Fatty Liver Disease"[Mesh]**

**OR diabet*[tiab]**

**OR prediabet*[tiab]**

**OR dysglyc*[tiab]**

**OR hyperglyc*[tiab]**

**OR insulin resist*[tiab]**

**OR metabolic syndrome[tiab]**

**OR obes*[tiab]**

**OR glucose intoleran*[tiab]**

**OR impaired glucose[tiab]**

**OR MASLD[tiab]**

**OR NAFLD[tiab]**

**OR "metabolic dysfunction-associated steatotic liver disease"[tiab]**

**OR "nonalcoholic fatty liver disease"[tiab]**

**OR "non-alcoholic fatty liver disease"[tiab]**

**)**

**AND**

**(**

**"Randomized Controlled Trial"[Publication Type]**

**OR "Controlled Clinical Trial"[Publication Type]**

**OR random*[tiab]**

**OR placebo*[tiab]**

**OR sham[tiab]**

**OR trial[tiab]**

**OR crossover[tiab]**

**OR cross-over[tiab]**

**)**

**AND 2000/01/01:2026/05/31[dp]**

**AND english[la]**

**Scopus**

**TITLE-ABS-KEY(**

**(**

**"fecal microbiota transplant*"**

**OR "faecal microbiota transplant*"**

**OR "fecal microbiome transplant*"**

**OR "faecal microbiome transplant*"**

**OR "fecal microbial transplant*"**

**OR "faecal microbial transplant*"**

**OR "stool transplant*"**

**OR "intestinal microbiota transfer*"**

**OR (transfer* AND intestinal AND microbiota)**

**OR "microbiota transfer therap*"**

**)**

**AND**

**(**

**diabet***

**OR prediabet***

**OR dysglyc***

**OR hyperglyc***

**OR "insulin resist*"**

**OR "metabolic syndrome"**

**OR obes***

**OR "glucose intoleran*"**

**OR "impaired glucose"**

**OR MASLD**

**OR NAFLD**

**OR "metabolic dysfunction-associated steatotic liver disease"**

**OR "nonalcoholic fatty liver disease"**

**OR "non-alcoholic fatty liver disease"**

**)**

**AND**

**(**

**random***

**OR placebo***

**OR sham**

**OR trial**

**OR crossover**

**OR "cross-over"**

**)**

**)**

**AND PUBYEAR > 1999**

**AND PUBYEAR < 2027**

**AND (LIMIT-TO(LANGUAGE, "English"))**

**Supplementary Table S2.** Glycaemic outcome availability, data preparation and inclusion in quantitative synthesis

| **Study** | **Selected outcome and assessment** | **Extracted n, FMT/control** | **Source and preparation of effect estimate** | **Role in synthesis** |
| --- | --- | --- | --- | --- |
| Vrieze et al., 2012 | Rd change at 6 weeks | 9/9 | Medians and ranges converted to means and SDs; change-score SD estimated using r=0.50 | Included in the descriptive lean-donor Rd synthesis |
| Kootte et al., 2017 | Rd change at 6 weeks | 26/12 | Medians and interquartile ranges converted to means and SDs; change-score SD estimated using r=0.50 | Included in the descriptive lean-donor Rd synthesis |
| Koopen et al., 2021 | HOMA-IR change at 6 weeks | 12/12 | Medians and interquartile ranges converted to means and SDs; change-score SD estimated using r=0.50 | Included in the primary HOMA-IR meta-analysis |
| de Groot et al., 2020 | Rd change at 2 weeks | 12/10 | Mean-change contrast and standard error digitised from the published figure reporting mean ± SEM | Presented separately according to donor metabolic phenotype |
| Yu et al., 2020 | HOMA-IR change through 12 weeks | 11/12 | Directly reported between-group mean difference and 95% CI | Included in the primary HOMA-IR meta-analysis |
| Gómez-Pérez et al., 2024 | HOMA-IR change at 12 weeks | 7/7 | Medians and interquartile ranges converted to means and SDs; change-score SD estimated using r=0.50 | Included in the primary HOMA-IR meta-analysis; HbA1c findings reported narratively |
| Wu et al., 2023 | HOMA-IR and HbA1c changes at 4 weeks | 9/12 | Mean changes reconstructed from arm-level means and SDs using r=0.50 | HOMA-IR included in the primary meta-analysis; HbA1c presented separately; baseline imbalance examined in influence analyses |
| da Ponte Neto et al., 2023 | HbA1c change at 6 weeks | 11/11 | Mean changes reconstructed from arm-level means and SDs using r=0.50 | Presented as an individual sham-controlled HbA1c contrast; not pooled |
| Mocanu et al., 2021 | HOMA2-IR change at 12 weeks | 34/36 | Mean changes reconstructed from arm-level means and SDs using r=0.50 | Excluded from the primary analysis; included only in the scale-mixed sensitivity analysis |
| Ng et al., 2022 | Fasting glucose, insulin and HbA1c through 24 weeks | — | Comparable between-group change estimates and standard errors were unavailable for the defined synthesis | Qualitative synthesis only |
| Allegretti et al., 2020 | Secondary glycaemic outcomes through 12–26 weeks | — | No eligible between-group HOMA-IR effect estimate was available | Qualitative synthesis only |
| Rinott et al., 2021 | Glycaemic outcomes during months 6–14 | — | The intervention evaluated diet-modulated autologous FMT during the weight-regain phase | Qualitative synthesis as a distinct clinical estimand |
| Smits et al., 2018 | Secondary metabolic outcomes at 2 weeks | — | No eligible between-group glycaemic effect estimate with sufficient dispersion data was available | Qualitative synthesis only |
| Su et al., 2022 | Fasting glucose and HbA1c at days 20 and 90 | — | Predominantly within-group and figure-based reporting, with post-randomisation attrition | Qualitative synthesis only |
| Groenewegen et al., 2025 | HOMA-IR change at 12 weeks | 10/10 | Direct longitudinal mixed-model contrast and 95% CI | Included in the primary HOMA-IR meta-analysis |
| Craven et al., 2020 | HOMA-IR change at 6 weeks | 15/6 | Between-group change estimate and corresponding standard error could not be derived from the available report | Eligible but not included in quantitative synthesis |
| Ghorbani et al., 2023 | HOMA-IR change at 3 months | 15/13 | Between-group change estimate and corresponding standard error could not be derived from the available report | Eligible but not included in quantitative synthesis |
| Piwchan et al., 2025 | HOMA-IR change at 6 weeks | 8/10 | Adjusted marginal-model contrast; standard error reconstructed from the reported 95% CI | Included in the primary HOMA-IR meta-analysis |
